# Medications To Treat Alcoholism

**Published:** 1999

**Authors:** Bankole A. Johnson, Nassima Ait-Daoud

**Affiliations:** Bankole A. Johnson, M.D., Ph.D., is a professor of psychiatry and pharmacology and deputy chairman for research and Nassima Ait-Daoud, M.D., is an instructor at the University of Texas Health Sciences Center, San Antonio, Texas

**Keywords:** drug therapy, AOD (alcohol and other drug) dependence, calcium acetylhomotaurinate, naltrexone, AOD craving, AOD abstinence, drug efficacy, antagonists, opioids, glutamate, serotonin, serotonin uptake inhibitors, dopamine, serotonin receptors, buspirone, combination drug therapy, patient compliance, literature review

## Abstract

Advances in neurobiology support the development of medications to treat alcoholism by modifying the activity of specific chemical messengers (i.e., neurotransmitters) in the brain. Among the most promising new medications is acamprosate, which appears to decrease the intensity of craving after a person has stopped drinking. Naltrexone (ReVia™) has been shown to decrease alcohol consumption, although its practical effectiveness may be compromised by poor patient compliance and other factors. Ondansetron shows promise for decreasing drinking and increasing abstinence rates among early onset alcoholics, who respond poorly to psychosocial treatment alone. Researchers are investigating whether the use of specific medications in combination can further enhance their effectiveness. Additional research is needed to determine how medications interact with different psychosocial factors and treatments.

Although psychosocial therapies help many alcohol-dependent persons reduce alcohol consumption and maintain abstinence, 40 to 70 percent of patients resume drinking within 1 year of such treatment (Swift 1999). Advances in neuroscience research in the past decade suggest the possibility of developing medications to improve the efficacy of concurrent psychological or behavioral addiction therapies (Litten et al. 1996). These advances include research that implicates specific neurotransmitter systems (see textbox, p. 100) in the development of addiction, suggesting that medications that modify the activity of these systems may interfere with the development of alcoholism. In addition, some alcoholics may be biologically predisposed to alcohol dependence. Those persons may benefit from medications designed to correct or ameliorate the biochemical abnormalities that presumably underlie their susceptibility.

This article reviews recent efforts to develop medications to both reduce the desire to drink and promote abstinence. Each section begins with a brief explanation of the probable role played by a specific neurotransmitter system in the development of addiction to alcohol and other drugs (AODs). These neurotransmitter systems include opioids, glutamate, serotonin (5HT), and dopamine. Medications that affect the function of these neurotransmitters to influence alcoholism are discussed, focusing on those medications that have shown the most promise in clinical trials on human subjects. Finally, the article recommends areas for future research.

## Opioid Antagonists

Opioid peptides are a class of neurotransmitters that produce physiological effects similar to those of morphine and heroin. In humans, opioid peptides modulate the effects of other neurotransmitters, thereby influencing a broad range of physiological functions (Froehlich 1997). Alcohol consumption affects the production, release, and activity of opioid peptides (Herz 1997). Opioid peptides appear to increase the rewarding effects of alcohol, nicotine, and opiates, contributing to the reinforcement of their use. Research suggests that this effect results from interaction with other neurotransmitter systems, particularly dopamine. For example, alcohol consumption by laboratory animals is reduced by naltrexone (ReVia^™^) and naloxone, antagonists of the mu receptor, one of the major subtypes of opioid receptor in the brain (Froehlich 1997). These medications have been shown to block the alcohol-induced release of dopamine in the nucleus accumbens (Swift 1999). Antagonists at the mu opioid receptor may also affect alcohol consumption by suppressing general consummatory behaviors without altering motivational or approach behaviors (for technical terms not defined in the text, see glossary, pp. 100) (Boyle et al. 1998). In addition, researchers (see Gianoulakis 1998) have proposed other mechanisms for the effects of opioid peptides on alcohol consumption, including modulation of the body’s hormonal stress response.

Human laboratory studies exploring the effects of naltrexone on alcohol-induced mood and craving have produced equivocal results (reviewed in Litten and Allen 1998). One factor that may contribute to the discrepancy among research results is a person’s genetic susceptibility to alcoholism. In a recent study (King et al. 1997), nonalcoholic social drinkers (average consumption of two drinks per day) consumed an alcoholic beverage 3 to 4 hours after taking naltrexone. Compared with subjects who did not have any alcoholic relatives, subjects with family histories of alcoholism (i.e., high-risk subjects) experienced less of alcohol’s stimulant effects but reported increased tension, fatigue, and confusion.

Consistent with the above results, persons at genetically high risk for developing alcoholism have lower levels of β-endorphin and demonstrate a more pronounced increase in β-endorphin levels in response to alcohol administration compared with persons who do not have alcoholic relatives. Similarly, naltrexone’s propensity to reduce alcohol intake in humans is greater in persons who have higher beta-endorphin levels (Gianoulakis et al. 1996).

The Food and Drug Administration has approved naltrexone for the treatment of alcohol dependence based largely on evidence from two randomized, double-blind, placebo-controlled studies. In the first of these studies (Volpicelli et al. 1992), recently abstinent male patients undergoing psychosocial alcoholism treatment who received 50 mg/day of naltrexone^1^ for 12 weeks reported less craving than subjects who received psychosocial treatment alone. In addition, the patients who received naltrexone maintained abstinence for longer periods and exhibited significantly reduced relapse rates than the patients who did not receive naltrexone (i.e., only 23 percent of naltrexone-treated patients compared with 54 percent of placebo-treated patients experienced relapse). Among patients who sampled alcohol while still in treatment, only 50 percent of the naltrexone-treated patients relapsed, compared with 95 percent of the patients who received a placebo (Volpicelli et al. 1992).

O’Malley and colleagues (1992) obtained comparable results in the second study using a similar design but including both male and female alcoholic subjects. An additional important finding in this study was the interaction between naltrexone use and the type of psychosocial therapy employed. The cumulative rate of abstinence was highest for patients who received naltrexone in conjunction with generalized supportive psychotherapy. However, the combination of naltrexone and a more intensive therapeutic program designed to teach specific coping skills was more effective at preventing relapse among patients who sampled alcohol.

**How Nerve Cells Communicate**

All mental and bodily functions are integrated by orderly communication among nerve cells (i.e., neurons). The ends of adjacent neurons are generally separated from one another by microscopic gaps called synapses. Specialized chemicals called neurotransmitters carry messages across the synapse from the “sending” (i.e., presynaptic) area of one neuron to the “receiving” (i.e., postsynaptic) area of another neuron. The message is “received” when the neurotransmitter locks onto a binding site (i.e., receptor protein) on the post-synaptic neuron. The binding of a neurotransmitter to its receptor causes a chemical change in the postsynaptic neuron, serving to stimulate, inhibit, or modulate a specific physiological function. A single neuron generally releases either only one or a few types of neurotransmitters but may contain receptors that bind to several different neurotransmitters. Under normal circumstances, neurotransmitters released into synapses are quickly removed, either by chemical degradation or by the action of transporter molecules, which carry the neurotransmitters back into the presynaptic neuron.

Further research has demonstrated that naltrexone-treated abstinent alcoholics report diminished craving for alcohol (O’Malley et al. 1996) and those who sample alcohol after a period of abstinence experience less mood elevation from the alcoholic beverage than non-naltrexone-treated patients (Volpicelli et al. 1995). Unfortunately, the protective effects of naltrexone appear to dissipate over time; O’Malley and colleagues (1996) found no difference in abstinence rates between naltrexone- and placebo-treated patient groups 5 months after naltrexone treatment was terminated.

The major obstacle to the practical clinical effectiveness of naltrexone appears to be poor patient compliance. In a 3-month followup study, Volpicelli and colleagues (1997) found modest improvement in alcohol consumption (measured as percent drinking days) only among patients who took their daily dose of naltrexone on at least 90 percent of the study days. The large-scale United Kingdom Collaborative study of Chick and co-workers (reviewed in Litten and Allen 1998) found no significant differences between naltrexone and the placebo on various measures of alcohol consumption over 12 months except among patients who exhibited at least an 80-percent compliance rate. The level of supervision necessary to ensure patient compliance may be difficult to achieve outside of specialized treatment facilities.

Further large-scale clinical trials are needed to establish naltrexone’s clinical effectiveness. Research also is needed to determine if naltrexone would be more effective at doses greater than 50 mg/day or if the treatment period were prolonged beyond 12 weeks of daily medication. In addition, particular subpopulations or types of alcoholics may be differentially responsive to naltrexone, especially alcoholics with strong family histories of alcoholism. For example, higher levels of craving measured when treatment was initiated were associated with greater treatment response (Jaffe et al. 1996), suggesting that naltrexone may be more effective in patients with the most severe alcoholism.

## Specific Glutamate Antagonists

The excitatory neurotransmitter glutamate, acting largely at the *N*-methyl-d-aspartate (NMDA) receptor,^2^ may contribute to some of alcohol’s effects (e.g., intoxication, cognitive impairment, and some symptoms of withdrawal^3^). The NMDA-receptor antagonist acamprosate appears to reduce the intensity of craving after drinking cessation, particularly when the patient is exposed to situations or environments associated with previous alcohol use, where the risk of relapse is greatest (Spanagel and Zieglgänsberger 1997).

Acamprosate has been shown to decrease alcohol consumption in rats without interfering with total fluid and food intake (Litten et al. 1996). Few laboratory studies have directly tested acamprosate’s effects on alcohol-related behaviors in humans. However, a magnetic resonance imaging (MRI) study showed reduced glutamatergic activity in certain brain regions in humans following acamprosate administration (Bolo et al. 1998), providing support for the therapeutic potential of acamprosate in the treatment of alcoholism.

Large-scale clinical studies support the effectiveness of acamprosate in treating alcoholism. An analysis of pooled data from a series of randomized, double-blind, placebo-controlled trials at several centers throughout Europe involving more than 3,000 alcoholic subjects indicated that twice as many patients remained abstinent from alcohol for up to 1 year after treatment compared with patients who received only a placebo (Swift 1999). For example, in a 12-week study, Lhuintre and colleagues (1990) showed that acamprosate (1.3 g/day) was more efficacious than placebo in the treatment of recently detoxified alcoholics, with the greatest improvement observed in the most severe alcoholics. Drawbacks of this study included the lack of standardized criteria for diagnosing alcoholism and the use of nonstandardized blood chemistry analyses to detect recent alcohol consumption.

In a similar study, Paille and colleagues (1995) found acamprosate at doses of both 2.0 g/day and 1.3 g/day to be superior to a placebo in the treatment of alcohol-dependent patients who drank an average of approximately 17 standard drinks per day. The percent of patients continuously abstinent was highest among those taking 2 g/day of acamprosate, lowest among those taking a placebo, and at an intermediate level among patients taking 1.3 g/day of acamprosate. The major improvements in this study, compared with that of Lhuintre and colleagues (1990), was the use of standardized diagnostic criteria to identify alcoholism and better laboratory standardization for monitoring alcohol consumption by blood chemistry, despite the relative insensitivity of such methods as an indicator of transient alcohol consumption.

In some trials, the 2-to-1 ratio of abstinence rates among acamprosate-treated patients compared with placebo-treated patients persisted for at least 1 year following cessation of treatment, although overall relapse rates had increased during the interval. At the end of 48 weeks of treatment, Sass and colleagues (1996) found abstinence rates of 43 percent among acamprosate-treated subjects compared with 21 percent among placebo-treated subjects. At the end of an additional 48 weeks without medication, abstinence rates were 39 percent and 17 percent, respectively (Sass et al. 1996). Similar results of effectiveness for acamprosate in the treatment of alcoholics have been reported by others (Whitworth et al. 1996; Geerlings et al. 1997).

## Serotonergic Medications

Serotonin plays a role in regulating diverse physiological functions, including body rhythms, sleep, and mood states, as well as appetitive and consummatory behaviors (Lovinger 1997). At least 15 subtypes of the serotonin receptor exist, some of which have potential roles in the development of alcohol-use disorders through multiple interactions with other neurotransmitter systems. The effects of serotonergic agents in the treatment of alcoholism have been well studied over the past two decades. This section examines the effects of general and specific serotonergic medications that show promise for alcoholism treatment.

### Selective Serotonin Reuptake Inhibitors

Selective serotonin reuptake inhibitors (SSRIs) are medications that interfere with the removal of serotonin from the synapse following release of the neurotransmitters (see textbox). Various SSRIs are used to treat psychiatric conditions, including mood disorders (e.g., depression) and anxiety disorders. The effects of SSRIs are not limited to specific serotonin-receptor subtypes.

Alcohol consumption has been shown to alter serotonin-receptor function in humans and animals, and early studies have indicated that administration of SSRIs can suppress alcohol consumption in animals (Lovinger 1997). Activation of specific serotonin-receptor subtypes can stimulate dopaminergic activity in the nucleus accumbens (see glossary, p. 106), thereby potentially contributing to alcohol’s rewarding effects (Lovinger 1997). However, the relationship between serotonin-reuptake inhibition, food intake, and fluid consumption is complex (Gill and Amit 1989). Thus, the effect of SSRIs on alcohol consumption may be attributable to generalized decreases in consummatory behavior rather than to a reduction in alcohol’s reinforcing effects (Swift 1999).

Despite encouraging results of animal studies (Lovinger 1997), the findings of clinical trials using SSRIs for the treatment of alcoholics without co-occurring psychiatric disorders have been equivocal. Although earlier studies showed that SSRIs can reduce alcohol consumption in alcohol abusers (Naranjo et al. 1990, 1992; Naranjo and Sellers 1989), more recent long-term studies have not confirmed these results (Gorelick and Paredes 1992; Kranzler et al. 1995).

Predicated on the findings of human laboratory studies showing that alcoholics with an early onset of disease may be deficient in serotonin (5HT) (Buydens-Branchey et al. 1989; Fils-Aime et al. 1996), Kranzler and colleagues (1996) examined the effects of the SSRI fluoxetine (Prozac^®^) on alcohol drinking among this subtype of alcoholic. The researchers presumed that fluoxetine would compensate for the functional deficit in early onset alcoholics, thereby resulting in a cessation of alcohol consumption. Surprisingly, however, Kranzler and colleagues (1996) found that fluoxetine appeared to increase alcohol consumption among early onset alcoholics, suggesting that the relationship between serotonergic dysfunction and the onset of alcoholism is neither simple nor direct.

Although a review of pharmacological agents for the treatment of alcoholics with co-occurring psychiatric disorders is outside the scope of this article, evidence suggests that SSRIs are effective for treating alcoholics with co-occurring depression.^4^ In the first controlled double-blind trial of an SSRI in alcoholics with co-occurring depression, Cornelius and colleagues (1997) found that both depressive symptoms and the level of alcohol consumption decreased significantly in patients who were administered fluoxetine compared with patients who were taking a placebo.

### Medications That Affect Specific Serotonin-Receptor Subtypes

#### Buspirone

Serotonin receptors of the subtype designated 5HT_1_ may be involved in general consummatory behavior as well as the development of alcohol intoxication and alcoholism (Lovinger 1997). Buspirone (Buspar^®^), which has partial agonist activity at the 5HT_1_ receptor, is commonly prescribed to treat anxiety disorders. Findings of reduced drinking in rats (Lovinger 1997) have led to clinical studies in human alcoholics. Results of controlled human studies are mostly negative, although some studies suggest that alcoholics treated with buspirone may exhibit some improvement in both craving and drinking behavior (reviewed in Malec and colleagues 1996). Evidence suggests that buspirone’s mechanism of action in reducing alcohol-related behaviors may result from reductions in anxiety, rather than through reduction in the rewarding effects of alcohol per se, and that any potential benefit from this medication is probably limited to alcoholics with independent co-occurring anxiety disorders (Malec et al. 1996).

#### Ritanserin

Serotonin 5HT_2_ receptors may play a role in alcohol’s rewarding effects and also contribute to the development of acute physical withdrawal symptoms (Lovinger 1997). Laboratory research demonstrates that certain 5HT_2_ antagonists can significantly suppress alcohol intake by rats without affecting water consumption, although some of these medications can affect food consumption (Myers and Lankford 1998; Overstreet et al. 1997). Antagonists at the 5HT_2_ receptor have also been shown to reduce alcohol intake by modulating the effects of dopamine in the nucleus accumbens (Lovinger 1997).

The effectiveness of ritanserin as a treatment for alcohol dependence was recently tested in a rigorous multicenter, placebo-controlled clinical trial in which 423 alcoholics received up to 5 mg/day concurrently with cognitive behavioral therapy. All study participants had significantly reduced their alcohol consumption by the end of the study period. However, no difference in drinking level was observed between patients receiving ritanserin and those who had received the placebo. Although higher ritanserin doses may have been more effective, high doses of 5HT_2_-receptor antagonists may increase the potential for adverse side effects, particularly irregular heartbeat (Johnson et al. 1996).

#### Ondansetron

The most promising serotonergic medication for treating alcoholism appears to be the 5HT_3_ antagonist ondansetron. Animal studies demonstrate that the 5HT_3_ receptor enhances the rewarding effects of alcohol, perhaps in part by increasing the alcohol-induced release of dopamine in the nucleus accumbens (Lovinger 1997). Consistent with this hypothesis, antagonists at the 5HT_3_ receptor have been shown not only to block dopamine activity but also to reduce alcohol intake in a variety of animals (for a review see Johnson and Cowen 1993).

In humans, ondansetron reduced some of alcohol’s positive subjective effects including self-reported desire to drink (Johnson and Cowen 1993; Swift et al. 1996). In a 6-week preliminary clinical trial, Sellers and colleagues (1994) found that 0.5 mg/day, but not 4 mg/day, was efficacious in treating moderate alcohol abusers. Researchers (Johnson et al. 1999) recently have presented a much larger double-blind clinical trial of ondansetron’s effectiveness as adjunctive treatment to cognitive-behavioral therapy in both early and late onset alcoholics. Preliminary analysis of results from that study showed that ondansetron was significantly superior to a placebo in reducing alcohol drinking and increasing abstinence rates among early onset but not late onset alcoholics. Previous data have shown that earlier age of onset for alcoholism is associated with certain biochemical measures thought to indicate serotonin dysfunction (Swann et al. 1999). This finding is consistent with literature positing serotonin dysfunction among early onset alcoholics (Buydens-Branchey et al. 1989; Linnoila and Virkkunen 1994). It is, therefore, plausible that ondansetron’s treatment effect may be associated with its ability to ameliorate serotonergic abnormality in this subtype of alcoholic (see figure below).

**Figure:**
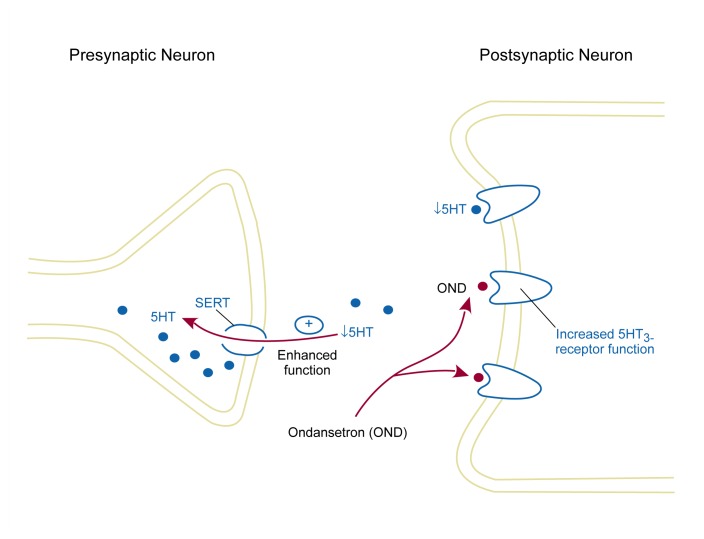
The proposed mechanism for the effectiveness of ondansetron (OND) in treating early onset alcoholics (EOAs). Serotonin (5HT) may increase the rewarding effects of alcohol through its actions at the 5HT_3_ receptor. EOAs may exhibit increased function of the serotonin transporter (SERT), a protein that helps remove 5HT from the synapse after its release. Therefore, 5HT levels in the synapse are reduced, which is consistent with findings of decreased serotonergic function in EOAs. Under these conditions, alcohol potentiates (i.e., upregulates) the activity of 5HT_3_ receptors. By blocking these upregulated 5HT_3_ receptors, OND may help restore normal serotonergic function.

Intriguingly, compounds that facilitate serotonin function, even though neurochemical analyses suggest a serotonin-deficient state, either have no effect or may worsen drinking behavior of early onset alcoholics (Kranzler et al. 1994*a*,*b*).

## Dopamine Antagonists

Although medications that directly affect dopamine function would appear hypothetically to be promising candidates for alcoholism treatment, experimental results have not been encouraging (Di Chiara 1997). In animals, a variety of dopamine antagonists, such as the antipsychotic medication haloperidol (Haldol^®^) and tiapride, were shown to attenuate low-dose alcohol-induced hyperactivity, whereas higher doses of alcohol were associated with sedation. In human alcoholics, tiapride was demonstrated in a randomized, placebo-controlled clinical trial to be superior to a placebo in increasing abstinence, self-esteem, and life satisfaction. Drinking levels also were significantly lower in the tiapride-using patients compared with the placebo group (Shaw et al. 1994). However, similar to other dopaminergic medications, an important problem with these medications is their potential to produce neurological side effects such as movement disorders (Tamion et al. 1990). Earlier research suggests that tiapride may be useful in alleviating alcohol-withdrawal symptoms (Swift 1999). However, tiapride may increase the likelihood of seizures, a common complication of withdrawal, and would probably need to be prescribed with anticonvulsants (Delmeire 1980). Thus, the side-effect profile of dopamine antagonists may limit their widespread use.

## New Vistas

### Opioid Antagonists

The mu receptor antagonist nalmefene has been shown to increase abstinence in a preliminary placebo-controlled, double-blind study of 21 alcoholics (Mason et al. 1994). Nalmefene is also less likely than naltrexone to produce the adverse side effect of liver damage. Consequently, researchers and clinicians anxiously await results of subsequent large-scale clinical trials of nalmefene in alcoholic populations.

Opioid-peptide receptors other than the mu receptor have been implicated in some alcohol-related behaviors (Herz 1997). Research suggests that antagonists of the delta opioid receptor (e.g., naltriben and naltrindole) may suppress alcohol consumption in animals (Froehlich et al. 1991). Further animal and human studies are needed.

### Calcium Channel Antagonists

Calcium has many physiological functions throughout the body and is required for nerve cell activity. Medications that interfere with the passage of calcium into cells (i.e., calcium channel blockers) are prescribed to treat high blood pressure and other cardiovascular conditions.^5^ Calcium channel blockers appear to ameliorate some symptoms of acute alcohol withdrawal, and some research suggests that these medications also may decrease alcohol consumption and reinforcement in animals (De Beun et al. 1996). In limited human research, however, the calcium channel blocker isradipine failed to alter self-reported subjective mood or hand-eye coordination associated with alcohol’s abuse liability (Rush and Pazzaglia 1998). Nevertheless, these results should not be taken as conclusive; thus, further studies are needed.

## Combination Treatments

Interactions among neurotransmitter systems suggest that combining therapeutic agents for the treatment of alcoholism may be an effective strategy. One potentially promising treatment would combine acamprosate and naltrexone. While naltrexone reduces craving derived from alcohol’s positive rewarding effects (Volpicelli et al. 1995), acamprosate diminishes craving associated with the period following acute withdrawal (Spanagel and Zieglgänsberger 1997). Thus, the combination of naltrexone and acamprosate might not only make it easier for a patient to abstain from alcohol use but also might prevent a “slip” from turning into a relapse. Several important issues must be resolved with any medication combination, however, including optimal dosing and timing, duration of treatment, and management of potential adverse interactions. NIAAA is currently funding clinical trials to establish the effectiveness of naltrexone and acamprosate, both alone and in combination, for the treatment of alcoholism.

## Conclusions

Of the medications reviewed, acamprosate’s potential appears to be the most widely established, although naltrexone remains the approved choice in the United States. Results of ongoing clinical trials suggest that naltrexone and acamprosate, both separately and in combination, may usher in a new era of medication treatments with multiple neurotransmitter targets. In addition, preliminary data suggest that ondansetron offers new hope for the treatment of early onset alcoholics, who generally tend to respond poorly to psychosocial treatment alone.

Nonetheless, important challenges remain. Based on the realization that alcoholics constitute varying subtypes with differing biological and psychosocial contributions to the disease, researchers must rigorously test medication development among these subgroups. Differential subgroup efficacy would not only provide an important therapeutic advantage to those who might benefit but also could offer information on the neurochemical factors mediating that aspect of the disease. Optimally, this would enable medications to be targeted toward the type of alcoholics who would most benefit from their use.

Additionally, few pharmacotherapy studies vary the dose of the medication studied. Without dose-ranging studies, the response ceiling may not be achieved nor would the benefits to adverse event relationship be characterized thoroughly. This could also lead to premature discarding of a potential therapeutic medication simply on the basis that the chosen dose was ineffective. Finally, the development of neuroimaging and molecular techniques promise new tools for both characterizing and charting the progress of disease and for understanding the site-specific actions of medications associated with treatment response. The coming decade promises more powerful tools for characterizing drug effects and for closing the gap between animal models of addiction and the human condition.

## Footnotes

1 This is the standard dose of naltrexone (under the brand name Trexan^®^) used for treating opiate addiction.

2 NMDA is a subtype of glutamate receptor, so named because it responds to the synthetic chemical agonist *N*-methyl-d-aspartate (NMDA).

3 Acute symptoms of withdrawal follow cessation of a period of heavy drinking and include tremors, profuse sweating, and seizures lasting several days. An unpleasant mental state may persist for months afterward, which the patient may try to alleviate by relapsing to drink.

4 Depressed mood can occur as a symptom of alcoholism. Each subject in this study met standard diagnostic criteria for co-occurrence of both alcoholism and a clinical depressive disorder independently.

5 Some calcium channel blockers used to treat high blood pressure include nifedipine (e.g., Adalat) and verapamil (e.g., Calan).

## GLOSSARY

Agonist: A drug or medication that mimics the effects of a *neurotransmitter* at a *receptor*.
Antagonist: A drug or medication that blocks or reverses the effects of a *neurotransmitter*.
Appetitive behavior: The activities involved in seeking and approaching a rewarding stimulus such as food or alcohol.
Consummatory behavior: The activities involved in contacting and obtaining a rewarding stimulus to attain its effects (i.e., eating or drinking).
Dependence: Addiction to alcohol (i.e., alcoholism) or to other drugs resulting in part from the effects of *reinforcement*.
Dopamine: A *neurotransmitter* that helps regulate *motivational* behavior, among other functions. Release of dopamine into the *nucleus accumbens* has been associated with the reinforcing effects of alcohol.
Endorphins: A class of *opioid peptides*, including beta-endorphin.
Glutamate: The primary excitatory *neurotransmitter* in the brain.
Motivational: Pertaining to mental states or behavior that increase the probability of approaching and attaining a reward (e.g., seeking and consuming alcohol). Motivational behavior includes *appetitive* and *consummatory* phases.
Negative craving: An urge or desire to resume alcohol consumption in response to neurological changes induced by heavy drinking.
Neuron: A nerve cell.
Neurotransmitter: A chemical messenger released by an excited or stimulated *neuron*. After release, *neurotransmitters* may travel across a *synapse* and then bind to a *receptor* on an adjacent neuron, usually triggering a series of chemical and electrical changes in the second cell.
Nucleus accumbens: A brain region affected by many drugs of abuse and implicated in the process of *reinforcement*.
Opioid peptides: *Neurotransmitters* with morphine-like effects, including mood elevation. Beta-endorphin and other *opioid peptides* may contribute to the development of alcoholism by modulating the activity of *dopamine* in the *nucleus accumbens*.
Partial agonist: An agent that is only partially effective as an agonist. Partial agonists also can act as *antagonists*.
Protein: The product of the genetic information encoded in a gene.
Receptor: A *protein* usually found on the surface of a *neuron* or other cell that recognizes and binds to *neurotransmitters* or other chemical messengers.
Reinforcement: A learning process that strengthens the ability of a rewarding stimulus to elicit *motivational* behavior.
Reward: The goal of motivated behavior, sometimes consciously perceived as a pleasurable state (e.g., the effects of mild alcohol intoxication) or as relief from a painful or uncomfortable state (e.g., amelioration of withdrawal symptoms).
Serotonin: A *neurotransmitter* that helps regulate multiple physiological functions, including *appetitive* and *consummatory behaviors*. Serotonin also may modulate the effects of *dopamine* in the *nucleus accumbens*.
Synapse: A microscopic gap separating adjacent *neurons* where *neurotransmitters* and *receptors* cluster.
Withdrawal: A syndrome that may occur following cessation or reduction of heavy drinking. Symptoms may include tremors, agitation, and seizures.

## References

[b1-arh-23-2-99] Bolo N, Nedelec JF, Muzet M, De Witte P, Dahchour A, Durbin P, Macher JP (1998). Central effects of acamprosate: Part 2. Acamprosate modifies the brain in-vivo proton magnetic resonance spectrum in healthy young male volunteers. Psychiatry Research.

[b2-arh-23-2-99] Boyle AEL, Stewart RB, Macenski MJ, Spiga R, Johnson BA, Meisch RA (1998). Effects of acute and chronic doses of naltrexone on ethanol self-administration in rhesus monkeys. Alcoholism: Clinical and Experimental Research.

[b3-arh-23-2-99] Buydens-Branchey M, Branchey M, Noumair D (1989). Age of alcoholism onset, I. Relationship to psychopathology. Archives of General Psychiatry.

[b4-arh-23-2-99] Cornelius JR, Salloum IM, Ehler JG, Jarrett PJ, Cornelius MD, Perel JM, Thase ME, Black A (1997). Fluoxetine in depressed alcoholics: A double-blind, placebo-controlled trial. Archives of General Psychiatry.

[b5-arh-23-2-99] De Beun R, Lohmann A, De Vry J (1996). Conditioned taste aversion and place preference induced by the calcium channel antagonist nimodipine in rats. Pharmacology, Biochemistry & Behavior.

[b6-arh-23-2-99] Delmeire F (1980). Tiapridal. Acta Psychiatrica Belgica.

[b7-arh-23-2-99] Di Chiara G (1997). Alcohol and dopamine. Alcohol Health & Research World.

[b8-arh-23-2-99] Fils-Aime ML, Eckardt MJ, George DT, Brown GL, Mefford I, Linnoila M (1996). Early-onset alcoholics have lower cerebrospinal fluid 5-hydroxyindoleacetic acid levels than late-onset alcoholics. Archives of General Psychiatry.

[b9-arh-23-2-99] Froehlich JC (1997). Opioid peptides. Alcohol Health & Research World.

[b10-arh-23-2-99] Froehlich JC, Zweifel M, Harts J, Lumeng L, Li TK (1991). Importance of delta opioid receptors in maintaining high alcohol drinking. Psychopharmacology.

[b11-arh-23-2-99] Geerlings PJ, Ansoms C, Van den brink W (1997). Acamprosate and prevention of relapse in alcoholics. European Addiction Research.

[b12-arh-23-2-99] Gianoulakis C (1998). Alcohol-seeking behavior. The roles of the hypothalamic-pituitary-adrenal axis and the endogenous opioid system. Alcohol Health & Research World.

[b13-arh-23-2-99] Gianoulakis C, de Waele JP, Thavundayil J (1996). Implication of the endogenous opioid system in excessive ethanol consumption. Alcohol.

[b14-arh-23-2-99] Gill K, Amit Z (1989). Serotonin uptake blockers and voluntary alcohol consumption. A review of recent studies. Recent Developments in Alcoholism.

[b15-arh-23-2-99] Gorelick DA, Paredes A (1992). Effect of fluoxetine on alcohol consumption in male alcoholics. Alcoholism: Clinical & Experimental Research.

[b16-arh-23-2-99] Herz A (1997). Endogenous opioid systems and alcohol addiction. Psychopharmacology.

[b17-arh-23-2-99] Jaffe AJ, Rounsaville B, Chang G, Schottenfeld RS, Meyer RE, O’Malley SS (1996). Naltrexone, relapse prevention, and supportive therapy with alcoholics: An analysis of patient treatment matching. Journal of Consulting & Clinical Psychology.

[b18-arh-23-2-99] Johnson BA, Cowen PJ (1993). Alcohol-induced reinforcement: Dopamine and 5-HT3 receptor interactions in animals and humans. Drug Development Research.

[b19-arh-23-2-99] Johnson BA, Jasinski DR, Galloway GP, Kranzler H, Weinreib R, Anton RF, Mason BJ, Bohn MJ, Pettinati HM, Rawson R, Clyde C (1996). Ritanserin in the treatment of alcohol dependence: A multi-center clinical trial. Psychopharmacology.

[b20-arh-23-2-99] Johnson BA, Roache JD, Javors MA, DiClemente CC, Cloninger CR, Prihoda T, Bordnick PS, Ait-Daoud N Ondansetron and alcohol consumption: Preliminary analysis of double-blind trial.

[b21-arh-23-2-99] King AC, Volpicelli JR, Frazer A, O’Brien CP (1997). Effect of naltrexone on subjective alcohol response in subjects at high and low risk for future alcohol dependence. Psychopharmacology.

[b22-arh-23-2-99] Kranzler HR, Burleson JA, Del Boca FK, Babor TF, Korner P, Brown J, Bohn MJ (1994a). Buspirone treatment of anxious alcoholics. A placebo-controlled trial. Archives of General Psychiatry.

[b23-arh-23-2-99] Kranzler HR, Kadden RM, Babor TF, Rounsaville BJ (1994b). Longitudinal, expert, all data procedure for psychiatric diagnosis in patients with psychoactive substance use disorders. Journal of Nervous & Mental Disease.

[b24-arh-23-2-99] Kranzler HR, Burleson JA, Korner P, Del Boca FK, Bohn MJ, Brown J, Liebowitz N (1995). Placebo-controlled trial of fluoxetine as an adjunct to relapse prevention in alcoholics. American Journal of Psychiatry.

[b25-arh-23-2-99] Kranzler H, Burleson J, Brown J, Babor T (1996). Fluoxetine treatment seems to reduce the beneficial effects of cognitive behavioral therapy in type B alcoholics. Alcoholism: Clinical and Experimental Research.

[b26-arh-23-2-99] Lhuintre JP, Moore N, Tran G, Steru L, Langrenon S, Daoust M, Parot P, Ladure P, Libert C, Boismare F (1990). Acamprosate appears to decrease alcohol intake in weaned alcoholics. Alcohol & Alcoholism.

[b27-arh-23-2-99] Linnoila M, Virkkunen M, Montgomery SA (1994). Psychopharmacology of depression. British Association for Psychopharmacology monograph.

[b28-arh-23-2-99] Litten R, Allen J (1998). Advances in the development of medications for alcoholism. Psychopharmacology.

[b29-arh-23-2-99] Litten RZ, Allen J, Fertig J (1996). Pharmacotherapies for alcohol problems: A review of research with focus on developments since 1991. Alcoholism: Clinical & Experimental Research.

[b30-arh-23-2-99] Lovinger DM (1997). Serotonin’s role in alcohol’s effects on the brain. Alcohol Health & Research World.

[b31-arh-23-2-99] Malec TS, Malec EA, Dongier M (1996). Efficacy of buspirone in alcohol dependence: A review. Alcoholism: Clinical & Experimental Research.

[b32-arh-23-2-99] Mason GA, Rezvani AH, Grady DR, Garbutt JC (1994). The subchronic effects of the TRH analog TA-0910 and bromocriptine on alcohol preference in alcohol-preferring rats: Development of tolerance and cross-tolerance. Alcoholism: Clinical & Experimental Research.

[b33-arh-23-2-99] Myers R, Lankford M (1998). Action of the 5-HT_2A_ antagonist amperozide on alcohol-induced poikilothermia in rats. Pharmacology, Biochemistry & Behavior.

[b34-arh-23-2-99] Naranjo CA, Sellers EM (1989). Serotonin uptake inhibitors attenuate ethanol intake in problem drinkers. Recent Developments in Alcoholism.

[b35-arh-23-2-99] Naranjo CA, Kadlec KE, Sanhueza P, Woodley-Remus D, Sellers EM (1990). Fluoxetine differentially alters alcohol intake and other consummatory behaviors in problem drinkers. Clinical Pharmacology & Therapeutics.

[b36-arh-23-2-99] Naranjo CA, Poulos CX, Bremner KE, Lanctot KL (1992). Citalopram decreases desirability, liking, and consumption of alcohol in alcohol-dependent drinkers. Clinical Pharmacology & Therapeutics.

[b37-arh-23-2-99] O’Malley SS, Jaffe AJ, Chang G, Schottenfeld RS, Meyer RE, Rounsaville B (1992). Naltrexone and coping skills therapy for alcohol dependence: A controlled study. Archives of General Psychiatry.

[b38-arh-23-2-99] O’Malley SS, Jaffe AJ, Rode S, Rounsaville BJ (1996). Experience of a “slip” among alcoholics treated with naltrexone or placebo. American Journal of Psychiatry.

[b39-arh-23-2-99] Overstreet D, McArthur R, Rezvani A, Post C (1997). Selective inhibition of alcohol intake in diverse alcohol-preferring rat strains by the 5-HT_2A_ antagonists amperozide and FG 5984. Alcoholism: Clinical and Experimental Research.

[b40-arh-23-2-99] Paille FM, Guelfi JD, Perkins AC, Royer RJ, Steru L, Parot P (1995). Double blind randomized multicentre trial of acamprosate in maintaining abstinence from alcohol. Alcohol and Alcoholism.

[b41-arh-23-2-99] Rush CR, Pazzaglia PJ (1998). Pretreatment with isradipine, a calcium-channel blocker, does not attenuate the acute behavioral effects of ethanol in humans. Alcoholism: Clinical & Experimental Research.

[b42-arh-23-2-99] Sass H, Soyka M, Mann K, Zieglgänsberger W (1996). Relapse prevention by acamprosate. Results from a placebo-controlled study on alcohol dependence [published erratum appears in the December 1996 issue of *Archives of General Psychiatry* 53(12):1097]. Archives of General Psychiatry.

[b43-arh-23-2-99] Sellers EM, Toneatto T, Romach MK, Somer GR, Sobell LC, Sobell MB (1994). Clinical efficacy of the 5-HT_3_ antagonist ondansetron in alcohol abuse and dependence. Alcoholism: Clinical & Experimental Research.

[b44-arh-23-2-99] Shaw GK, Waller S, Majumdar SK, Alberts JL, Latham CJ, Dunn G (1994). Tiapride in the prevention of relapse in recently detoxified alcoholics. British Journal of Psychiatry.

[b45-arh-23-2-99] Spanagel R, Zieglgänsberger W (1997). Anti-craving compounds for ethanol: New pharmacological tools to study addictive processes. Trends in Pharmacological Sciences.

[b46-arh-23-2-99] Swann AC, Johnson BS, Cloninger CR, Chen YR (1999). Relationships of plasma tryptophan availability to course of illness and clinical features of alcoholism: A preliminary study. Psychopharmacology.

[b47-arh-23-2-99] Swift RM (1999). Drug therapy for alcohol dependence. New England Journal of Medicine.

[b48-arh-23-2-99] Swift RM, Davidson D, Whelihan W, Kuznetsov O (1996). Ondansetron alters human alcohol intoxication. Biological Psychiatry.

[b49-arh-23-2-99] Tamion F, Petit J, Massari P, Leroy J, Biga N, Oksenhendler G (1990). Malignant Neuroleptic Syndrome during tiapride treatment. Journal de Toxicologie Clinique et Experimentale.

[b50-arh-23-2-99] Volpicelli JR, Alterman AI, Hayasgida M, O’Brien CP (1992). Naltrexone in the treatment of alcohol dependence. Archives of General Psychiatry.

[b51-arh-23-2-99] Volpicelli JR, Watson NT, King AC, Sherman CE, O’Brien CP (1995). Effect of naltrexone on alcohol “high” in alcoholics. American Journal of Psychiatry.

[b52-arh-23-2-99] Volpicelli JR, Rhines KC, Rhines JS, Volpicelli LA, Alterman AI, O’Brien CP (1997). Naltrexone and alcohol dependence. Role of subject compliance. Archives of General Psychiatry.

[b53-arh-23-2-99] Whitworth AB, Fisher F, Lesch OM, Nimmerrichhter A, Oberbauer H, Platz T, Potgieter WH, Fleischhaker WW (1996). Comparison of acamprosate and placebo in long-term treatment of alcohol dependence. Lancet.

